# Advances in Pathophysiology and Novel Therapeutic Strategies for Coronary No-Reflow Phenomenon

**DOI:** 10.3390/biomedicines13071716

**Published:** 2025-07-14

**Authors:** Hubert Borzuta, Wiktor Kociemba, Oliwia Bochenek, Monika Jarowicz, Agnieszka Wsół

**Affiliations:** 1 Chair and Department of Experimental and Clinical Physiology, Laboratory of Centre for Preclinical Research, Medical University of Warsaw, Banacha 1b, 02-097 Warsaw, Poland; hubert.borzuta@wum.edu.pl (H.B.); s085113@student.wum.edu.pl (W.K.); s081029@student.wum.edu.pl (M.J.); 2 Międzylesie Specialist Hospital in Warsaw, Bursztynowa 2, 04-749 Warsaw, Poland; oliwia5457@wp.pl

**Keywords:** coronary no-reflow, myocardial infarction, animal models, pathophysiology, ischemia/reperfusion injury, microvascular obstruction

## Abstract

Coronary no-reflow (CNR) is the failure of blood to reperfuse ischemic myocardial tissue after restoration of the vasculature. CNR poses a significant clinical challenge in the treatment of patients with ST-segment elevation myocardial infarction (STEMI), as it increases mortality and the risk of major adverse cardiac events (MACEs). Myocardial ischemia with subsequent reperfusion results in severe damage to the cardiac microcirculation. The pathophysiological causes of CNR include cardiomyocyte vulnerability, capillary and endothelial damage, leukocyte activation, reactive oxygen species (ROS) production, and changes in microRNA profiles and related gene expression. The impact of percutaneous coronary intervention (PCI) on the occurrence of CNR cannot be overlooked, as it can provoke distal atherothrombotic embolization. Current standards of pharmacological therapy for CNR are confined to intracoronary vasodilators and antiplatelet agents. As our understanding of the pathogenesis of the CNR phenomenon improves, opportunities emerge for developing novel therapeutic strategies. The following literature review provides an overview of the pathophysiology of the no-reflow phenomenon (based on animal and preclinical studies), contemporary treatment trends, and current therapeutic approaches.

## 1. Introduction

Cardiovascular diseases (CVD) remain the leading cause of global mortality, with ischemic heart disease (IHD) accounting for almost half of all deaths. In 2022, IHD affected approximately 315 million people worldwide and resulted in over 9 million deaths [1]. Furthermore, the number of disability-adjusted life years (DALYs) reached 182 million, contributing to rising healthcare costs [2]. The most severe manifestation of IHD is acute coronary syndrome (ACS), with approximately 3 million people annually affected by ST-segment elevation myocardial infarction (STEMI), and 4 million diagnosed with non-ST-segment elevation myocardial infarction (NSTEMI) [3]. The gold standard for the treatment of acute STEMI is percutaneous coronary intervention (PCI) [4]. The Euro Heart Survey on Acute Coronary Syndrome II study revealed that the in-hospital mortality rate for patients with ACS was 4.4% overall and 5.3% for STEMI, while the 30-day mortality rate was 5.6% overall and 6.4% for STEMI [5]. Despite a reduction in door-to-balloon time to less than 90 min, in-hospital mortality did not change significantly [6]. This observation underscores the urgent need for the development of supplementary therapeutic strategies and highlights the potential influence of additional factors on patient prognosis, with the coronary no-reflow (CNR) phenomenon being a salient candidate.

CNR is a dreaded complication of PCI, which is the failure of blood to reperfuse an ischemic area of myocardial tissue after restoration of the vasculature. Beyond the heart, the no-reflow phenomenon can manifest in brain, kidney, and patches of skin [7,8,9]. The prevalence of CNR ranges from 2% to 44% of all patients undergoing PCI, and mortality ranges from 7.4% to 30.3% due to varying definitions and criteria [10]. The most common clinical scenario for CNR is STEMI and the typical anatomic setting is a complex, thrombus-burdened lesion, specifically when it is located in a large coronary artery. CNR may also occur after stent placement in simple lesions. This is especially true for atherectomy techniques or angioplasty in saphenous vein grafts, as well as final attempts at optimization techniques with larger balloons and/or higher pressures [11]. Moreover, post-PCI CNR in NSTEMI patients is frequently linked to periprocedural myocardial infarction, a complication that significantly increases the risk of mortality in both the short and long term [12].

A variety of both invasive and non-invasive techniques are utilized to evaluate the presence of a CNR phenomenon or microvascular obstruction (MVO). These include <50% or <70% ST-segment resolution (depending on the cut-off used) in electrocardiographic recordings at 60 to 90 min after reperfusion in patients with STEMI, TIMI (thrombolysis in myocardial infarction) flow grade 0–2, myocardial blush grade (MBG) 0 to 1, and, finally, measurements of flow or resistance parameters: coronary flow reserve (CFR) with a value less than 2.0 or post-procedural microvascular resistance index (IMR) values > 25 units, suggesting microcirculation obstruction [11,13,14,15,16]. The most sensitive technique for detecting CNR is contrast-enhanced cardiac magnetic resonance (CMR). Microvascular obstruction (MVO) appears as an absence of both early and late gadolinium enhancement [17]. Notably, the lack of late contrast uptake strongly correlates with adverse cardiac remodeling and worse clinical outcomes [18,19]. The prediction of CNR can be further improved by utilizing tools that rely on biochemical markers, such as the ALBI score, which is derived from serum albumin and total plasma bilirubin levels [20].

**Figure 1:**
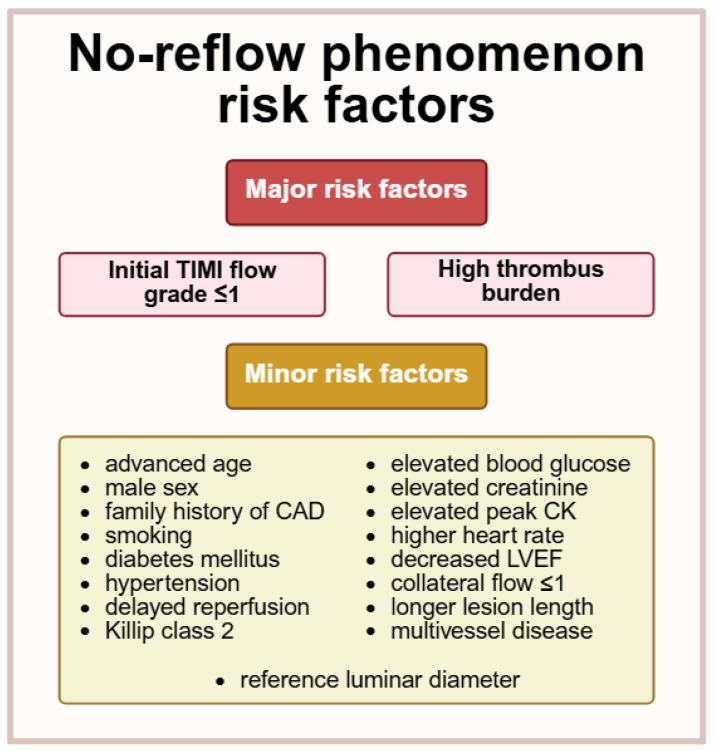
No-reflow phenomenon risk factors. TIMI—thrombolysis in myocardial infarction, CAD—coronary artery disease, CK—creatine kinase, LVEF—left ventricular ejection fraction. Created in BioRender. Borzuta, H. (2025) https://BioRender.com/q86cdtq, accessed on 9 July 2025.

In a meta-analysis of 27 studies, several risk factors were associated with CNR, the most prominent being initial TIMI flow grade ≤ 1 (OR 3.83, 95% CI [2.77–5.29], *p* < 0.0001) and high thrombus burden (OR 3.69, 95% CI [2.39–5.68], *p* < 0.0001) [10]. Figure 1 provides a comprehensive overview of all other minor risk factors (OR < 3.5) identified in this meta-analysis [10]. Recently, Dawson et al. proposed the NORPACS (no-reflow prediction in acute coronary syndrome) risk scale based on an analysis of a population of more than 30,000 patients [21]. Six variables were included in the model: presence of cardiogenic shock, STEMI with symptom onset > 195 min before PCI, estimated stent length ≥ 20 mm, vessel diameter < 2.5 mm, TIMI flow grade < 3 before PCI, and lesion location (left main coronary artery or bypass graft). Scoring ≥8 was associated with >20% suboptimal coronary reperfusion in the proposed model [21]. Measuring atherosclerotic plaque with optical coherence tomography (OCT) is a novel and promising method for CNR risk stratification. Decreased fiber cap thickness, as measured by OCT, has been identified as an independent risk factor for CNR [22].

The occurrence of CNR is associated with a deterioration in short- and long-term clinical outcomes. The clinical consequences of CNR are highly variable, ranging from a brief episode of mild angina with transient ST-segment changes to a prolonged episode of chest pain with refractory ST-segment elevation or hypotension [11]. Even within the catheterization laboratory, it can lead to sudden hemodynamic decompensation, malignant ventricular arrhythmias, cardiogenic shock, or death [23]. In the long term, CNR increases the risk of cardiac death, all-cause mortality, malignant ventricular arrhythmias, and congestive heart failure [24]. These deleterious sequelae are attributable to a failure of healing in the damaged myocardium, the presence of a more tenuous scar and an augmented infarct size [25].

The objective of this review is to shed light on the recent advancements in understanding the pathophysiology of CNR, which may provide a framework for the development of novel targeted therapeutic strategies. Furthermore, the text provides a comprehensive overview of the animal models and techniques employed in preclinical studies of CNR. Finally, we review emerging preclinical and clinical therapeutic approaches that have shown encouraging results in addressing CNR.

## 2. Animal Models of Coronary No-Reflow

**Figure 2:**
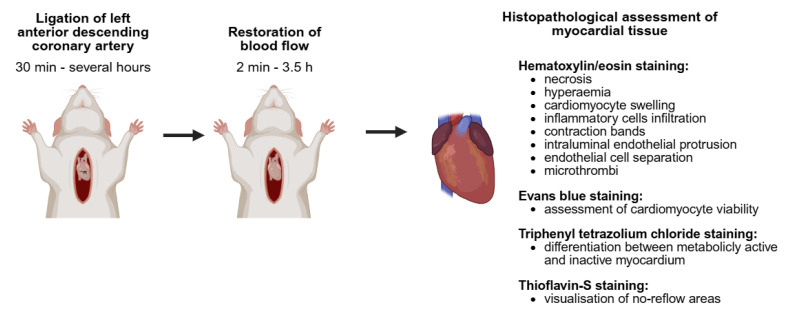
Establishment of a CNR animal model and histopathological characterization of myocardial injury. Created in BioRender. Borzuta, H. (2025) https://BioRender.com/iowv8mx, accessed on 9 July 2025.

The expansion of knowledge regarding the pathophysiology of cardiovascular diseases and the development of novel therapeutic strategies necessitate extensive preclinical research. In vivo studies are of particular significance in this context, as they facilitate the assessment of functional parameters, consider the complex structure of the heart, and allow for the evaluation of animal survival following various therapeutic interventions. Several animal models have been developed to gain a deeper insight into the pathophysiological processes that contribute to the emergence of CNR (Figure 2).

The most commonly utilized animal species or strains are Sprague–Dawley rats, Fisher rats, Guangxi Bama minipigs, Gottingen minipigs, dogs and rabbits [25,26,27,28,29,30,31,32,33,34,35,36]. CNR may be induced by ligation of the left anterior descending coronary artery for a period of 30 min to several hours, followed by the restoration of blood flow [25,26,27,28,34,35,36]. As demonstrated by Kloner et al., the risk of developing CNR increases in proportion to the duration of ischemia [34]. In a canine model of ischemia/reperfusion (I/R) injury, CNR occurred exclusively after 90 min of coronary artery occlusion, but not after 40 min [34]. Reperfusion time also exerts a significant influence on the development of microcirculation damage. In the study by Ambrosio et al., the area of impaired flow exhibited a threefold increase between 2 min and 3.5 h following reperfusion [35]. In another study, Reffelmann et al. demonstrated that the area of CNR primarily increases during the initial 2 h of reperfusion, with only minor changes observed between 2 and 8 h of restored flow [36].

An alternative approach to mimicking CNR is MVO using microspheres. In rodent models, approximately 3000 microspheres suspended in saline are injected into the left ventricle during a 10 to 15 s occlusion of the ascending aorta, thereby ensuring that the microspheres enter the coronary vessels [32,37,38]. In contrast, in the swine model, microspheres are administered directly into the left anterior descending coronary artery via a catheter inserted through the femoral artery [30]. The targeted administration of microspheres more accurately reflects ACS in humans, as typically only one coronary vessel is affected. The induction of MVO can also be achieved by using sodium laurate in combination with a 30 s aortic arch occlusion. However, it should be noted that only approximately 50% of rats exposed to sodium laurate developed CNR [29].

Various techniques are used to evaluate the effectiveness of the aforementioned models in inducing CNR. It has been demonstrated that pigs or rats subjected to I/R injury or MVO exhibit elevated serum troponin levels and impaired cardiac systolic function, assessed by echocardiography [30,32]. Histopathological assessment of the myocardium reveals the presence of foci of necrosis, characterized by the loss of cell nuclei. In addition, the tissue in close proximity to areas of ischemia displays a swelling, hyperemia and infiltration by inflammatory cells [30,32]. The areas of immediate CNR are characterized by the presence of cardiomyocyte coagulation necrosis with cellular edema, but without contraction bands. In contrast, a myocardium that experienced initial flow demonstrates endothelial damage and the formation of contraction bands in the cardiomyocytes [35]. In the coronary microcirculation, intraluminal endothelial protrusion and endothelial cell separation can be observed, while the capillary lumen is frequently obliterated by tightly packed red blood cells, with adjacent platelets and thrombi [34]. When microspheres are used, they can be visualized within blocked arterioles.

Several types of staining are employed to assess the perfusion and metabolic activity of cardiomyocytes. Evans blue staining is a valuable tool for the assessment of cardiomyocyte viability [28]. Triphenyl tetrazolium chloride (TTC) is used as a redox indicator to differentiate between metabolically active and inactive regions [31]. Finally, the administration of thioflavin-S intravenously prior to animal sacrifice enables the visualization of no-reflow areas within TTC-negative tissue [26,27,31]. The part of the myocardium where blood flow disturbances are most frequently observed is the subendocardium, whereas the subepicardium is rarely affected. Coronary blood flow and areas of CNR can also be visualized in a living animal using isotope-labelled ammonia ^13^N-NH_3_ in positron emission tomography (PET) [39,40]. An alternative method for evaluating regional myocardial blood flow is the administration of radioactive microspheres prior to animal sacrifice. The radioactivity of the tissue is then quantified in relation to the radioactivity of the blood. Regions where blood flow is compromised exhibit diminished radioactivity [25].

## 3. Pathophysiology of Coronary No-Reflow

**Figure 3:**
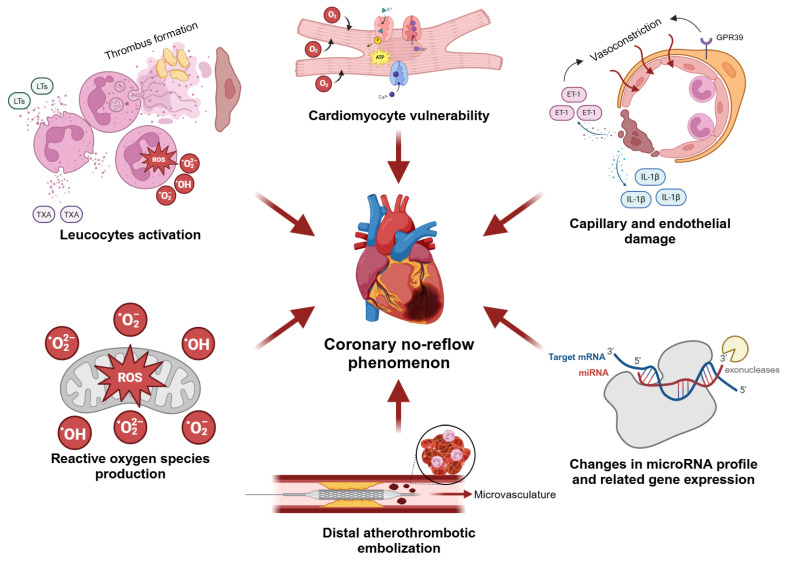
The summary of pathomechanisms underlying the development of CNR. LTs—leukotrienes, TXA—thromboxane A, ROS—reactive oxygen species, ATP—adenosine triphosphate, ET-1—endothelin-1, IL-1β—interleukin-1β, GPR39—G-protein coupled receptor 39. Created in BioRender. Borzuta, H. (2025) https://BioRender.com/zs3vs9e, accessed on 9 July 2025.

Several pathophysiological causes of CNR have been identified, including cardiomyocyte vulnerability, distal atherothrombotic embolization, and capillary and endothelial damage. In addition, leukocyte activation, reactive oxygen species (ROS) production, and changes in microRNA profile appear to be critical contributors to the initiation and propagation of cardiac microvascular pathologies (Figure 3).

### 3.1. Cardiomyocyte Vulnerability

A number of intrinsic properties of cardiomyocytes and myocardial tissue render them particularly susceptible to I/R injury and microcirculation disturbances that result in the development of CNR. First of all, cardiomyocytes are highly differentiated cells and therefore have a significantly reduced potential for regeneration. Although I/R injury usually results in their death by necrosis, more structured forms of death have also been observed, including apoptosis, autophagy, necroptosis, pyroptosis, pyrolysis and ferroptosis [41,42,43,44]. Moreover, myocardial tissue remains highly susceptible to edema and the accumulation of water both intracellularly and interstitially. This is due to the dense microvasculature and the high interstitial flow rate [45]. A 3% increase in extravascular fluid represents a 12% increase in total cardiac weight and results in a 30% decrease in cardiac output [46]. The osmolality of a normal cardiomyocyte is approximately 305 mOsm/kg. However, during hypoxia, this value increases, reaching a maximum of 420 mOsm/kg after 70−90 min of ischemia, which is caused by the accumulation of both ions and other osmotically active substances [47]. The myocardium has a high metabolic rate, relying mainly on oxidative phosphorylation, and is very efficient at extracting oxygen at a level of 70%−80% [48]. Ischemia leads to a rapid decline in cellular adenosine triphosphate (ATP) levels due to a shift to anaerobic oxidation, which inhibits the function of vital ion pumps such as Ca^2+^-ATPase and Na^+^/K^+^-ATPase [49,50]. This leads to increased intracellular levels of Na^+^ and Ca^2+^. Consequently, edema develops and phospholipases are activated, exacerbating ionic disturbances via cell membrane damage [51]. Increased cellular Ca^2+^ levels are also consistent with the histological findings of contractile band necrosis. During reperfusion, there is a restoration of ATP levels, and the contraction of cardiomyocytes is rapid, which in turn leads to cellular damage [47]. Endothelial damage also appears to contribute to cellular edema and swelling. It has been established that myocardial edema exhibits a bimodal pattern, with the initial peak occurring at reperfusion and dissipating after 24 h, and the subsequent peak emerging at 7 days after I/R [52]. The initial wave of edema is directly associated with reperfusion, as described by the aforementioned mechanisms. In contrast, the second wave is believed to be a consequence of tissue regeneration and immune cell infiltration, as evidenced by the observation that steroid therapy reduces the water content in the ischemic myocardium [53].

Reperfusion, although appearing to be the salvation for ischemic tissue, causes more damage than hypoxia itself [54]. The rapid restoration of blood flow creates a pH difference which pulls the H^+^ ions out of the cells and creates a massive Na^+^ influx. This in turn produces a reverse Na^+^/Ca^2+^ portal action and results in a massive intracellular Ca^2+^ overload, which causes rapid cardiomyocyte contraction and contraction band necrosis as seen histologically [55].

### 3.2. Distal Atherothrombotic Embolization

Distal atherothrombotic embolization is another proposed mechanism for CNR, and is consistent with the finding that a high thrombus burden remains a risk factor for the development of CNR. Distal embolization has been found to occur principally subsequent to stenting, and is the result of mechanically induced detachment of thrombus fragments, which then occlude microcirculation vessels. Coronary macroembolization contributes to MVO, potentiating inflammation and vasoconstriction. Furthermore, the release of partial debris and inflammatory substances from the culprit epicardial lesion may sensitize the coronary arteries to angina, even in the absence of major obstruction [56]. Microvascular embolisms are comprised of platelets and fibrin, with ischemic patients exhibiting heightened platelet activity due to elevated platelet 4 factor (PF4) and catecholamine concentrations [57]. These embolisms may also contain red blood cells, neutrophils, lymphocytes and cholesterol crystals [58]. The lymphocyte count has been observed to increase over time, whilst the platelet count has been shown to decrease, a finding that is consistent with the presence of lymphocyte infiltrations around the ischemic tissue. On the other hand, the neutrophil and monocyte counts remained consistent at both 3 and 6 h of reperfusion [59,60]. Coronary microembolization is also associated with increased levels of tumor necrosis factor alpha (TNF-α) and sphingosine. The NO-TNF-α-sphingosine signaling pathway contributes to a gradual decline in contractile function [61].

Angiographically visible distal embolization was found in 13.4% of STEMI patients [62]. Risk factors that increased the probability of angiographically visible distal embolism were elevated blood glucose on admission, erythrocyte-rich thrombus, vessel diameter ≥ 3.5 mm, and pre-balloon dilation [62]. A much higher prevalence of distal atherothrombotic embolization was reported by Okamura et al., where embolic particles were found in 35 of 37 patients [63]. This finding correlated with an initial reduction in TIMI flow rate [63]. However, in a multicenter randomized clinical trial, embolic protection did not result in greater reperfusion success, smaller infarct size or improved survival [64]. Deferred stenting was a therapeutic approach employed to mitigate the risk of distal atherothrombotic embolization in the DANAMI-3 DEFER trial. This strategy reduced the incidence of CNR when compared with the conventional PCI. The benefits were particularly pronounced in older patients and in those with total coronary occlusion or a high thrombus burden [65]. 

### 3.3. Capillary and Endothelial Damage 

Damage to the cardiac capillaries has been proposed as the main pathology underlying the CNR phenomenon. In transmission electron microscopy imaging, the endothelium of areas with impaired blood flow exhibits signs of swelling and is characterized by a reduced number of pinocytic vesicles, clumped nuclear chromatin, and the protrusion of endothelial cells into the vessel lumen [34,66]. Disruption of endothelial integrity leads to an accumulation of interstitial fibrin deposits and erythrocyte extravasation [34]. Although these changes occur before blood flow is restored and are therefore caused by ischemia, reperfusion exacerbates them [34,35]. No association has been demonstrated between systemic endothelial function and the occurrence of CNR in patients with STEMI. Moreover, the development of CNR is probably not influenced by pre-existing coronary endothelial dysfunction [67].

The regeneration of ischemic myocardium depends on the preservation of vascular integrity. In areas of CNR, endothelial integrity is impaired, resulting in increased microvascular permeability. In rats subjected to I/R injury, the expression of vascular endothelial cadherin (VE-cadherin), vascular endothelial growth factor receptor 2 (VEGFR2), β-catenin, and γ-catenin was reduced in endothelial cells in regions with impaired blood flow, whereas mRNA transcription of these proteins was increased in areas with restored flow [68,69,70]. The loss of adherens junctions proteins leads to microvascular leakage. This dysfunction was further exacerbated in angiopoietin-like 4 gene knockout mice, while the use of losartan or carvedilol preserved adherens junctions and reduced the extent of CNR [68,69,70]. The laminin receptor (LR) also plays an important role in maintaining vascular integrity. LR inhibits endothelial cadherin (E-cadherin) internalization by binding to TIMAP/PP1c. Post-translational modifications of LR, such as dephosphorylation at Y47 and T125, are more important in limiting the no-reflow phenomenon than increased LR expression [40].

Vascular integrity is further compromised by the loss of endothelial cells. I/R injury activates caspase-4, leading to inflammation and endothelial cell death by pyroptosis [71]. Beclin-1 and histone H3 lysine 9 demethylase (KDM3A) help reduce endothelial pyroptosis [71,72]. Consequently, infarct size and the no-reflow zone decrease, and infiltration by macrophages and neutrophils is reduced [71]. However, KDM3A knockdown in CMECs impairs cell proliferation and migration, as well as reducing angiogenic potential [72].

Alongside endothelial cells, pericytes are an integral part of the microcirculation. Pericytes are cells located along the capillary walls that regulate microvascular flow and permeability. They also participate in inflammation, fibrosis, and angiogenesis, contributing to the development of the no-reflow phenomenon [73]. Pericytes act as the final defensive layer against decreased myocardial perfusion by constricting capillaries during ischemia to maintain adequate perfusion pressure. Their contraction is triggered by an increase in Ca^2+^ concentration resulting from ischemia, as well as the activation of the orphan receptor GPR39 [74,75]. While this response is protective during ischemia, prolonged pericyte contraction during reperfusion impairs blood flow. In rats, ischemia preconditioning attenuated I/R-induced pericyte contraction, concurrently reducing infarct size and CNR area [28]. Similar outcomes were achieved by knocking down the GPR39 receptor or the administration of VC43-specific GPR39 inhibitor. These interventions also increased the cardiac capillary density and diameter [75].

The regulation of microcirculatory tension is also mediated by endothelin (ET), a group of vasoactive peptides that cause capillary constriction by acting through ET-A and ET-B receptors. Elevated plasma levels of ET-1 in patients with STEMI correlated with an increased incidence of CNR and increased mortality within the first year after MI and in the long term [76,77,78]. Furthermore, increased ET-1 expression acted as independent predictor of CNR on CMR imaging [77]. In addition to its vasoconstrictive effects, ET stimulates the migration of inflammatory cells into the ischemic zone. By activating ET-A receptors on neutrophils, endothelin stimulates the production of platelet-activating factor (PAF) and promotes neutrophil adhesion to endothelial cells [79,80].

The enhanced interactions between leukocytes and the endothelium are also attributable to the loss of glycocalyx. The glycocalyx is a layer formed by the extracellular domains of various molecules associated with the endothelial cell membrane [81]. Ischemia has been shown to induce only minor local damage to the glycocalyx, which increases at the time of reperfusion. This damage is at least partially caused by the hydroxyl radical generated in the Fenton reaction [82]. The disruption of the glycocalyx’s ultrastructure not only stimulates neutrophil adhesion, but also contributes to increased vascular resistance in the microcirculation as a consequence of the loss of NO-mediated vasodilatory function of the endothelium [81,83].

In conclusion, the robust function of the coronary microcirculation relies on intact endothelial cell junctions, functional pericytes, a balance between vasoconstricting and vasodilating agents, and undamaged glycocalyx. Any alterations in any of these factors contribute to the development of CNR.

### 3.4. Role of Leucocytes 

Another pathomechanism underlying CNR is progressive leukocyte activation and capillary plugging. Engler et al. reported that the frequency of leukocytes in no-reflow myocardial capillaries was ten times that in sufficiently perfused myocardium [84]. Researchers suggested that leukocyte plugging of capillaries has a significant impact on the development of CNR during the first hour of myocardial ischemia [85]. It was also demonstrated that the extent of the CNR zone in dogs was diminished in the neutropenic group when compared to the group with a normal neutrophil count [86]. Capillary obstruction by neutrophils not only constitutes a mechanical obstruction to flow, but also results in the release of potent vasoactive mediators (e.g., thromboxanes, leukotrienes) and proteolytic enzymes, as well as the generation of ROS. The effects of this cascade of events are a significant obstruction to flow, the degeneration of smooth muscle and endothelial cells, and the destabilization of the atherosclerotic plaque [85,87,88]. Moreover, mounting evidence suggests a pathological role for neutrophil extracellular traps (NETs) induced during ischemia. Ge et al. hypothesized that NET formation provides a scaffold for thrombosis and may exacerbate endothelial damage [89]. Platelets accumulating in the CNR area also release thromboxane A2, platelet derived growth factor, serotonin, lipoxygenase products, proteases, and adenosine, which alter polymorphonuclear cell activation [90]. The increased inflammatory response results in platelet accumulation and subsequent coronary thrombus formation.

### 3.5. Role of Reactive Oxygen Species

The oxidative burst is a process linked to cellular reperfusion injury. It begins immediately after reperfusion and peaks as early as 10 s after blood flow is restored [91]. The production of oxygen free radicals during I/R overloads the cellular antioxidant defense, leading to oxidative stress. Increased ROS levels cause vascular dysfunction and thrombus formation in arterioles, impaired perfusion, increased capillary fluid leakage, and augmented fluid and protein outflow [92]. Additionally, ROS promote adhesion between blood cells and the endothelium of postcapillary veins. Potential sources of ROS in the heart during reperfusion include mitochondria, several isoforms of nicotinamide adenine dinucleotide phosphate (NADP), oxidase (NOX), xanthine oxidase (XOR), uncoupled endothelial nitric oxide synthase (eNOS), and infiltrating neutrophils [88,93,94,95,96]. However, the main source of oxidants remains unclear.

It was hypothesized that during ischemia, components of the mitochondrial respiratory chain are maximally reduced, and that during reperfusion, they release electrons onto oxygen to form superoxide [97]. However, Pisarenko et al. found that tissue levels of alanine, malate and succinate accumulate during MI due to the near-stoichiometric consumption of glutamate and aspartate [98]. These levels then decrease rapidly after reperfusion [98]. Since then, evidence has emerged that mitochondrial ROS production originates primarily from complex I via succinate-driven reverse electron transport [99,100]. This was further supported by blocking complex I with rotenone, which reduced ischemic damage to cardiolipin, cytochrome c and cytochrome oxidase [101]. Nevertheless, other factors like mitochondrial swelling and membrane rupture should also be considered as contributors to mitochondrial ROS production.

The NOX family consists of seven members, NOX1-5 and DUOX1-2, which use NAPDH as an electron donor and catalyze its transfer to oxygen to produce superoxide [102,103]. The role of NOX in oxidative injury remains controversial, as animal models have yielded conflicting results [104,105,106]. NOX has been implicated in neovascularization, cardiac remodeling and fibrosis. As noted by Sirker et al., there are significant differences between particular isotypes, as well as between humans and rodents [103]. Additionally, the NOX2 signaling pathway and ROS production have been shown to regulate mitochondrial fission in coronary endothelial cells in a mouse model of cardiac I/R injury [95].

Boueiz et al. indicated XOR as another source of ROS contributing to CNR [93]. Several XOR inhibitor-based strategies were evaluated in myocardial I/R injury models, but no protective effects were observed [107]. Nevertheless, the interaction between XOR and NOS is noteworthy, as NOS has also been implicated as another source of ROS. It is currently suspected that NOS is involved in XOR inactivation. Increased XOR activity in pulmonary endothelial cells was observed after the administration of NOS inhibitors, while XOR activity decreased after supplementation with L-arginine or the administration of nitric oxide (NO) donors [108]. In aortic endothelial cells subjected to hypoxia and reoxygenation, XOR-induced ROS production caused tetrahydrobiopterin (BH4) depletion. Since BH4 is a cofactor for eNOS, its depletion caused eNOS uncoupling and superoxide production instead of NO [109].

Excessive ROS production significantly impacts clinical outcomes. It is associated not only with increased infarct size and a higher risk of developing CNR, but also with a poorer long-term prognosis, including an increased risk of ventricular tachyarrhythmias and heart failure.

### 3.6. Role of microRNAs

Recently, increasing evidence has suggested an important role for microRNA molecules in the pathophysiology of several cardiovascular diseases, including the CNR [110]. MicroRNAs (miRNAs, miRs) constitute an evolutionary-conserved class of small non-coding RNAs of 19 to 25 nucleotides in size, which exert a negative regulatory effect on post-transcriptional gene expression [111,112]. MiRNAs participate in the regulation of a multitude of biological processes, including cell growth and differentiation, embryogenesis, intercellular communication, apoptosis, and epigenetic modifications [113]. The abundance of miRNAs (approx. 1900 molecules registered in miRBase) and their isomers (isomiRs), coupled with imperfect pairing with the mRNA sequence, allows for the silencing of more than half of protein-coding genes in humans [114].

**Figure 4:**
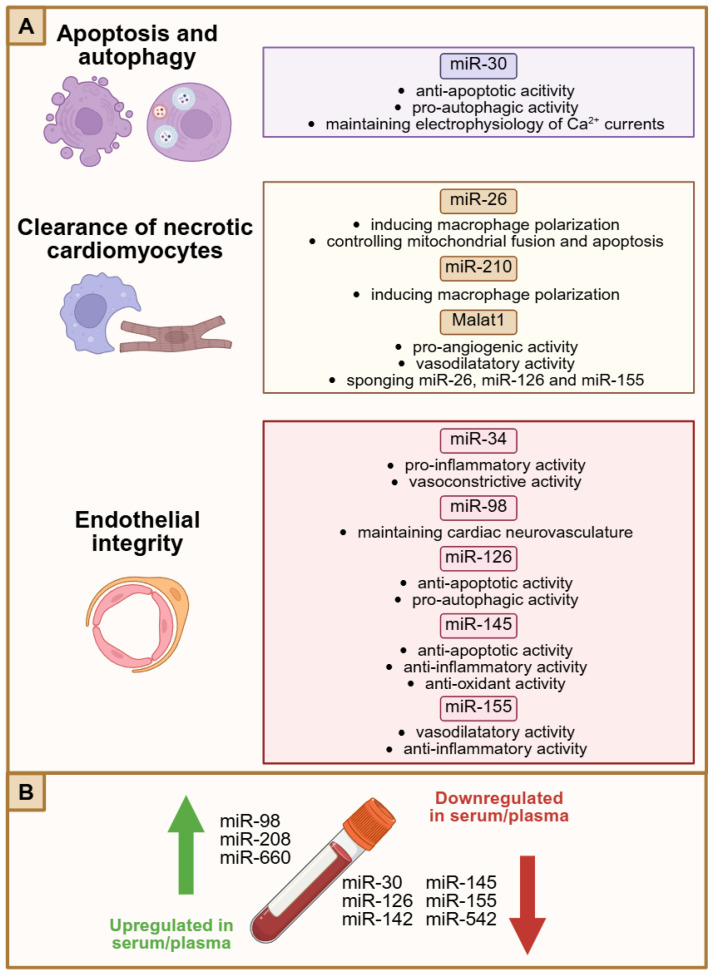
Role of miRNAs in the pathophysiology of CNR. (**A**) Several miRNAs have been found to regulate cardiomyocyte apoptosis and autophagy, facilitate clearance of necrotic cardiomyocytes and maintain endothelial integrity during I/R injury. (**B**) MiRNAs may serve as potential biomarkers in prediction of CNR, as some of them are upregulated or downregulated in serum or plasma in patients with CNR. Created in BioRender. Borzuta, H. (2025) https://BioRender.com/m1k77bh, accessed on 9 July 2025.

Several miRNAs are involved in the pathophysiology of myocardial I/R injury and the development of CNR (Figure 4), of which miR-30 has been the most extensively studied. MiR-30 plays a significant role in the regulation of apoptosis and autophagy. In human induced pluripotent stem cell-derived cardiomyocytes (hiPSC-CMs) subjected to 24 h of hypoxia, a reduction in the expression of miR-30 was observed. This finding correlated with a significant increase in caspase-3 activity and the extent of apoptosis, as well as a disruption in the electrophysiology of Ca^2+^ currents, which manifested as a reduction in amplitude and a prolongation of the latency period [115]. The use of an miR-30 analog has been demonstrated to reverse hypoxia-induced changes in hiPSC-CMs by inhibiting the expression of BIM, a known inducer of apoptosis and suppressor of autophagy [115]. A reduction in the expression of miR-30 was also observed in the myocardial tissue of rats that underwent coronary microembolization [32,116]. Transmission electron microscopy revealed an elevated number of autophagocytic vesicles in myocardium at 3 h post-ischemia, which subsequently declined at 9 h. The expression levels of the apoptosis-regulating proteins LC3-II and p62 exhibited corresponding changes, with an increase and decrease, respectively, observed after 3 h, and a subsequent decrease and increase after 9 h [32]. It seems that miR-30 exerts its effects on autophagy and apoptosis through the indirect regulation of the transcription factor Egr-1 [116]. In clinical studies, plasma miR-30 concentrations were reduced in STEMI patients with CNR compared to those with complete reperfusion [117,118,119]. Decreased miR-30 levels were linked to reduced left ventricular ejection fraction (LVEF) and elevated hs-CRP levels [119]. Furthermore, reduced miR-30 concentration may serve as an independent risk factor and predictor of CNR in patients presenting with STEMI [117,119]. Decreased miR-30 levels in patients with CNR were also associated with elevated TUG1 long noncoding (lncRNA) and NPPB mRNA expression. The TUG1/miR-30/NPPB reciprocal axis has been proposed as a nomogram model for predicting the occurrence of CNR after PCI [118].

As outlined above, the clearance of necrotic cardiomyocytes following I/R injury facilitates the regeneration of damaged tissue and scar formation. Extracellular vesicles obtained from cardiosphere-derived cells (CDCs) have been demonstrated to induce macrophage polarization towards a phenotype that is more efficient at removing apoptotic vesicles [120]. This process was associated with the inhibition of a disintegrin and metalloprotease domain 17 (ADAM17), resulting in the stimulation of the MerTK signaling pathway by the presence of miR-26 and miR-210 in the CDCs-derived vesicles [120]. Moreover, miR-26 seems to be regulated by Malat1 lncRNA in cardiomyocytes, where miR-26 affects mitochondrial fusion and apoptosis by modulating the expression of Mfn1 [121]. The pivotal role of MALAT1 in microvascular function, angiogenesis and vasodilation was confirmed by knocking down this lncRNA in mice subjected to MI. This silencing was associated with reduced survival, increased myocardial remodeling, decreased microvascular density, and lower levels of eNOS and VEGFR2 expression [121]. Additionally, Malat1 has been observed to sponge other miRNAs, including miR-126 and miR-155. Plasma levels of miR-126 and miR-155 were reduced after PCI in patients with CNR compared to those with normal reflow [117]. The noncanonical miR-126 pathway was activated under conditions of ischemia or high shear stress, thereby maintaining endothelial integrity. This protection was achieved by reducing caspase-3 activity, inducing autophagy and inhibiting apoptosis [122,123]. Conversely, in patients with hemodynamically insignificant coronary stenosis, plasma miR-155 levels were elevated in the slow-flow group in comparison to those with normal perfusion [124].

The successful reperfusion of the microvasculature is contingent upon endothelial homeostasis, whereby miR-98, miR-145 and miR-34 exert a pivotal influence [29,125,126,127]. Endothelial cells are a likely source of circulating miR-98, which has been verified to target NGF mRNA. The serum levels of miR-98 were elevated in patients with CNR, which was associated with greater incidence of major adverse cardiovascular events (MACEs) following MI [126]. The administration of a miR-98 antagonist to rats has been demonstrated to enhance cardiac function and diminish infarct size in I/R-induced microvascular dysfunction [126]. Moreover, the process of ageing was found to impair the neurovasculature of the heart. A decline in microvascular and axonal density was linked to a reduction in miR-145 and an increase in semaphorin-3A expression [125]. Similarly, diminished miR-145 expression was found in the serum of patients with CNR. Cardiac microvascular endothelial cells (CMECs) exposed to miR-145 analog in hypoxia/reoxygenation conditions were protected against apoptosis, oxidative stress and inflammation via the regulation of SMAD activity [127]. On the other hand, in rats subjected to coronary microembolization, pretreatment with an miR-34 analog resulted in unfavorable outcomes, whereas miR-34 antagonist alleviated cardiac dysfunction. The silencing of miR-34 activated the Sirt1/eNOS and inhibited the Sirt1/NFκB signaling pathways, which contributed to vasodilation and the attenuation of the inflammatory response in the myocardium, respectively [29].

Several other miRNAs have been linked to the occurrence of CNR. Elevated levels of miR-208 and miR-660, in tandem with reduced concentrations of miR-142 and miR-542 in serum or plasma, have been associated with a greater incidence of CNR following PCI in STEMI patients [30,128,129,130]. Therefore, the aforementioned miRNAs may serve as biomarkers in the prediction of CNR in clinical practice.

## 4. Novel Therapeutic Strategies for Coronary No-Reflow—Preclinical Studies

The advancement in comprehension of the CNR phenomenon’s pathophysiology has paved the way for the development of novel therapeutic strategies. Currently, extensive preclinical research is being conducted to evaluate the efficacy of several pharmacological compounds, medical interventions and cell therapies (Table 1).

In recent years, there has been considerable focus on the development of treatments aimed at reducing the release of ROS and protecting mitochondria. Researchers have demonstrated that applying a specific inhibitor of ROS production at the IQ site of complex I of the mitochondrial respiratory chain—OP2113—results in a reduction in mitochondrial ROS (mtROS) generation, accompanied by a reduction in infarct size and CNR area [27,131]. It was found that OP2113 should be administered as early as possible to reduce infarct size. However, a similar relationship was not found for the CNR phenomenon. Regardless of whether OP2113 was administered therapeutically or prophylactically, a reduction in the CNR area was achieved [27]. 

Another compound that has been demonstrated to reduce mtROS generation is anisodamine [132]. Anisodamine activates the mitochondrial K_ATP_ channels, which are closed under normal metabolic conditions. Opening of K_ATP_ channels causes an increase in K^+^ conductance, which stabilizes the resting membrane potential, and also shortens the action potential and reduces Ca^2+^ influx. The result is preservation of intracellular energy stores and prevention of Ca^2+^ overload, which protects the mitochondria from exceeding their own antioxidant capacity [133]. Moreover, anisodamine increases the content of metabolic index ATP and the activity of superoxide dismutase (SOD), while improving the ultrastructure of myocardial tissue. In the study conducted on isolated hearts, anisodamine enhanced hemodynamic parameters and reduced the infarct area. Application of the mitoK_ATP_ specific blocker, 5-hydroxydecanoic acid, led to the inhibition of the above effects [132]. Similar effects were obtained by using pinacidil—a non-selective blocker of K_ATP_ channels [134].

As previously mentioned, eNOS is listed among the potential sources of ROS during reperfusion injury. However, it is also known that eNOS induces the synthesis of NO, which plays an important role in regulating vascular tension. During I/R, the cAMP/PKA pathway leads to eNOS phosphorylation, which in turn stimulates its activity [135]. Rui Chen et al. reported that using the Tongmai Yangxin pill (TMYX) reduces the area of CNR via endothelium-dependent NO-cGMP signaling [136]. The TMYX has been found to induce NO synthesis and subsequent microvessel relaxation. In another study, it was shown that TMYX can enhance the anti-oxidative stress capabilities and reduce cell apoptosis [137]. However, it is still unclear which chemical components in this pill are responsible for its therapeutic effect.

In a study by Dai et al., a rat model of I/R injury was subjected to post-reperfusion therapeutic hypothermia initiated 1 min after reperfusion [138]. The results of this study revealed that hypothermia reduced the CNR area by more than 50% compared with the normothermia group. Moreover, it has been reported that even when hypothermia is initiated 30 min after reperfusion, it still results in a reduction in the extent of no-reflow [139]. However, no beneficial effect on the risk area for ischemia and infarct size was observed in either experiment. A possible explanation for this phenomenon is that hypothermia helps preserve the endothelial barrier and reduce inflammation, thereby mitigating MVO, but it does not impact already necrotized myocardial tissue. Further research is necessary to evaluate this hypothesis.

Another strategy to reduce CNR by inhibiting oxidative stress is hydrogen gas inhalation. In a rat I/R injury model, the total levels of ROS and the myocardial tissue MDA were reduced by 47.5% and 37.3%, respectively, in the group that received hydrogen inhalation compared to the control group [140]. Among the multiple mechanisms of ROS-induced microvascular dysfunction is the initiation of NLRP3-mediated endothelial cell pyroptosis [141]. The inhalation of hydrogen gas has been observed to have an anti-pyroptotic effect, as evidenced by the decreased expression of key proteins and enzymes associated with the pyroptosis, including NLRP3, ASC, caspase-1, GSDMD, and IL-1β [140].

Luteolin has also been proven to exert protective effects against I/R injury and CNR via inhibition of the TLR4-mediated NF-κB/NLRP3 inflammasome pathway. Administration of luteolin resulted in reduced ROS generation and attenuated cardiac inflammatory response [142]. Moreover, it has been demonstrated to maintain endothelial integrity by stimulating the Wnt/β-catenin pathway [39].

Diabetes mellitus serves as a well-established risk factor for the development of both IHD and CNR [143]. In patients with diabetes, there are multiple systemic and myocardial pathophysiological changes that make them more susceptible to microvascular dysfunction. Chronic hyperglycemia contributes to reduced endothelium-dependent vascular relaxation in response to vasodilators. Moreover, the majority of patients diagnosed with type 2 diabetes mellitus (T2DM) exhibit hypoadiponectinemia [144]. Administration of exogenous adiponectin to rats with T2DM one week before myocardial I/R resulted in an improvement in endothelium-dependent vasorelaxation and a decrease in CNR area. The endothelium-protective properties of adiponectin are attributed to its anti-inflammatory, antioxidant, and anti-apoptotic effects [145].

Among cell therapies, the intracoronary administration of cardiosphere-derived cells (CDCs) merits special attention in CNR treatment. CDCs are cardiac stem cells that support the regeneration of damaged myocardium by generating new cardiac tissue, paracrine activity, and secreting exosomes [146]. In animal models, intracoronary administration of CDCs has been shown to reduce the infarct size and the CNR area, while improving functional parameters and alleviating adverse myocardial remodeling [146,147,148]. A decrease in MI size and the no-reflow area was noted when CDCs were given either 5 min before or 30 min after reperfusion [147,148]. However, only pre-reperfusion administration preserved left ventricular ejection fraction (LVEF) [148]. In contrast, Vakrou et al. reported that intracoronary delivery of CDCs at the onset of reperfusion did not reduce microvascular obstruction or provide lasting functional improvement [149].

The ongoing preclinical investigation into diverse strategies for the prevention and treatment of the no-reflow phenomenon offers promise for the development of therapeutic approaches applicable in clinical practice. However, it is essential to acknowledge the inherent limitations and translational barriers of studies conducted in vitro and in vivo. The pharmacokinetic profiles of the studied compounds may vary considerably in human subjects. Furthermore, these agents modulate intracellular signaling pathways, wherein excessive activation or inhibition may elicit undesirable effects. Notably, the therapeutic efficacy of many of these substances is only exhibited when administered prior to the onset of myocardial infarction, which presents clear limitations in clinical settings.

**Table 1:** The summary of novel therapeutic approaches for the treatment of myocardial reperfusion injury in preclinical studies.

Drug/Therapeutic Intervention	Molecular Mechanisms of Action	Pharmacological Effects	Ref.
OP2113	*Inhibition of ROS formation at site I_Q_ of complex I of the mitochondrial respiratory chain;* Increased ATP production; Increased mitochondrial affinity to oxygen.	Reduced no-reflow zone size and MI area; No effect on blood pressure and heart rate.	[27,131]
Anisodamine	*Opening mitochondrial K_ATP_ channels;* Reduced ROS generation; Increased ATP production; Improved ultrastructure of myocardial tissue.	Increased LVDP and coronary flow; Reduced MI area; Reduced occurrence of ventricular reperfusion arrhythmias after I/R.	[132]
Pinacidil	*Nonselective opening of K_ATP_ channels;* Inhibition of calcium overload-induced mitochondrial dysfunction; Reduced cardiomyocyte and endothelial apoptosis; Increased NO activity and reduced levels of ET-1.	Reduced no-reflow zone size and MI area; Maintained endothelial barrier integrity; Improved left ventricular function; Attenuated left ventricular remodeling.	[134]
Tongmai Yangxin pill	*Activation of cAMP/PKA and NO/cGMP signaling pathways;* *Activation of Nrf2/HO-1 and inhibition of p38-MAPK signaling pathways;* Increased NO activity; Reduced ROS generation; Reduced cardiomyocyte apoptosis.	Relaxation of coronary microvessels; Reduced no-reflow zone size and MI area; Reduced infiltration of inflammatory cells and interstitial edema; Improved left ventricular function.	[136,137]
Post-reperfusion therapeutic hypothermia		Reduced no-reflow zone size; No impact on MI area.	[138,139]
Hydrogen gas inhalation	*Inhibition of oxidative stress and NLRP3-mediated endothelial pyroptosis;* Improved ultrastructure of myocardial tissue.	Reduced no-reflow zone size and MI area; Improved left ventricular function.	[140]
Luteolin	*Inhibition of TLR4/NF-κB/NLRP3 inflammasome pathway;* *Activation of Wnt/β-catenin pathway;* Reduced ROS generation; Attenuated cardiac inflammatory response.	Reduced no-reflow zone size and MI area; Increased R-amplitude in ECG; Improved left ventricular function; Maintained endothelial barrier integrity.	[39,142]
Adiponectin	*Improvement in endothelium-dependent vasodilatation;* Reduced levels of ET-1, ICAM-1 and VCAM-1.	Reduced no-reflow zone size; Improved left ventricular function.	[145]
Intracoronary administration of CDCs ^a^	*Formation of new cardiac tissue;* Reduced cardiomyocyte apoptosis.	Reduced no-reflow zone size and MI area; Improved left ventricular function; Attenuated left ventricular remodeling.	[146,147,148,149]
BSF 461314, bosentan, tezosentan, BQ 123	*Endothelin receptors ET-A and ET-B antagonists;* Attenuated cardiac inflammatory response; Inhibition of neutrophils adhesion and activation.	Reduced no-reflow zone size and MI area; Maintained endothelial barrier integrity; No impact on left ventricular function.	[79,83,150,151]

^a^ Conflicting data on the effectiveness of the therapeutic intervention. ROS—reactive oxygen species, ATP—adenosine triphosphate, MI—myocardial infarction, LVDP—left ventricular developed pressure, I/R—ischemia/reperfusion, NO—nitric oxide, ET-1—endothelin-1, cAMP—cyclic adenosine monophosphate, PKA—protein kinase A, cGMP—cyclic guanosine monophosphate, Nrf2—nuclear factor erythroid 2-related factor 2, HO-1—heme oxygenase-1, MAPK—mitogen-activated protein kinase, NLRP3—NOD-, LRR- and pyrin domain-containing protein 3, TLR4—toll-like receptor 4, NF-κB—nuclear factor kappa-light-chain-enhancer of activated B cells, ECG—electrocardiogram, ICAM-1—intercellular adhesion molecule 1, VCAM-1—vascular cell adhesion molecule 1, ET-B—endothelin-B, CDCs—cardiosphere-derived cells.

## 5. Novel Therapeutic Strategies for Coronary No-Reflow—Clinical Studies

Multiple therapeutic pharmacological and non-pharmacological strategies have been used to prevent and treat CNR and refractory CNR. Based on the results of several studies, intracoronary vasodilators such as adenosine, calcium channel blockers and nitrates, used alone or in combination, and antiplatelet agents such as glycoprotein IIb/IIIa (GP) inhibitors, are the mainstay of treatment for CNR [11,15,152,153,154]. Among non-pharmacological strategies, evidence supports the use of thrombectomy, distal protection devices, selective intracoronary injection of saline solution through a thrombus aspiration catheter and deferred stenting [11,15,155,156].

Recently, the treatment of CNR refractory to other therapies with epinephrine has shown encouraging results [157,158,159,160,161]. Epinephrine has well-known β-1 agonist properties with inotropic and chronotropic stimulation of the myocardium, but at low doses, its potent β-2 receptor agonist properties mediate vasodilatation of the arteriolar bed [162]. The beneficial effects of intracoronary epinephrine administration in refractory CNR cases in terms of significant improvement in coronary flow were first described by Skelding et al. [157]. Specifically, improved coronary flow was observed in 93%, and TIMI 3 flow was restored in 69%. Later reports from the RESTORE observational study showed that intracoronary epinephrine during primary PCI in STEMI patients with refractory CNR who failed conventional therapy (nitrates, thrombectomy, GP IIb/IIIa inhibitors, and adenosine) significantly improved coronary flow patterns and reduced the 30-day composite of death or heart failure [158]. The results of the above mentioned studies are supported by the most recent findings of Ryabov et al., where intracoronary administration of epinephrine during PCI in STEMI patients with refractory CNR is more effective than conventional treatments [159]. This approach improves coronary flow in the infarct-related artery, facilitates faster resolution of STEMI, improves ejection fraction (effect observed only in the epinephrine arm) and reduces MVO volume with a safety profile comparable to conventional treatment strategies. Compared to intracoronary adenosine, intracoronary epinephrine demonstrated significantly higher TIMI flow and lower final TIMI frame count frequency in the single-center COAR trial [160]. The other study provided evidence that local delivery of epinephrine with verapamil or adenosine to the distal coronary bed (via microcatheter or perfusion catheter) was more effective in the treatment of CNR than their guided catheter-delivery. However, no difference between epinephrine with verapamil and adenosine has been reported [161].

A recently published pilot study of excimer laser coronary angioplasty (ELCA) evaluated the efficacy of administering a cocktail consisting of a solution containing nitroglycerin, heparin, and verapamil as part of CNR prevention during ECLA. The study found that, in a total of 54 procedures, the incidence of CNR was significantly lower in the cocktail group (0% vs. 17.9%). Of course, a multicenter study with larger sample sizes is needed to confirm the above results [163].

In recent years, the possibility of using alprostadil (prostaglandin E1, PGE1) has also been a subject of discussion. A meta-analysis of 18 clinical trials (1458 patients) suggests that PGE1 reduces the incidence of microcirculatory dysfunction in MI [164]. Nevertheless, further research in this area is required to provide more robust evidence.

## 6. Conclusions

CNR is a phenomenon that complicates up to 40% of primary PCIs in acute MI. It is associated with unfavorable evolution of the disease, higher incidence of MACEs and worse patient prognosis. The etiopathogenesis of CNR is complex and results from the coexistence of several pathologies, in particular capillary and endothelial damage, cardiomyocyte vulnerability, distal atherothrombotic embolization, leukocyte activation, ROS production, and alterations in the miRNA profile. Although it is a common clinical problem with serious health implications, there are still no highly effective, targeted therapies. Therefore, novel therapeutic strategies that reduce ROS production, exert a protective effect on the endothelium or facilitate the regeneration of damaged myocardium are being evaluated in preclinical studies. It seems that due to the multifactorial etiopathogenesis and heterogeneous clinical presentation, the therapeutic strategy should be tailored to the given clinical circumstances, using both pharmacological and non-pharmacological treatments in order to improve patients’ prognosis. We believe that operator experience, which remains underestimated in the available literature, is also of great importance in the management of CNR.

## Data Availability

Data sharing is not applicable.
